# A Clip Domain Serine Protease Involved in Egg Production in *Nilaparvata lugens*: Expression Patterns and RNA Interference

**DOI:** 10.3390/insects10110378

**Published:** 2019-10-30

**Authors:** Jia-min Wu, Rong-er Zheng, Rui-juan Zhang, Jin-liang Ji, Xiao-ping Yu, Yi-peng Xu

**Affiliations:** Zhejiang Provincial Key Laboratory of Biometrology and Inspection & Quarantine, China Jiliang University, Hangzhou 310018, China; wujiamin94@163.com (J.-m.W.);

**Keywords:** *Nilaparvata lugens*, proclotting enzyme, RNAi, follicular cell, egg production

## Abstract

Clip domain serine proteases play vital roles in various innate immune functions and in embryonic development. *Nilaparvata lugens* proclotting enzymes (*Nl*PCEs) belong to this protease family. *NlPCE1* was reported to be involved in innate immunity, whereas the role of other *NlPCEs* is unclear. In the present study, *N. lugens proclotting enzyme-3* (*NlPCE3*) was cloned and characterized. *Nl*PCE3 contains a signal peptide, a clip domain, and a trypsin-like serine protease domain. *NlPCE3* was expressed in all tissues examined (gut, fat body, and ovary), and at all developmental stages. Immunofluorescence staining showed that *Nl*PCE3 was mainly expressed in the cytoplasm and cytomembrane of follicular cells. Double stranded *NlPCE3* RNA interference clearly inhibited the expression of *NlPCE3*, resulting in abnormal egg formation and obstruction of ovulation. These results indicate that *Nl*PCE3 plays an important role in egg production in *N. lugens*.

## 1. Introduction

The brown planthopper (BPH), *Nilaparvata lugens*, is one of the most harmful rice crop pest insects throughout Asia and causes tremendous economic losses upon emergence [1]. *N. lugens* easily develops resistance to pest-resistant rice varieties and insecticides, making it hard to effectively control. To a large extent, this is ascribed to *N. lugens* being an R-selected species with high fertility and strong invasive capacity [2]. Therefore, genes involved in ovarian development or oogenesis are attractive targets for controlling *N. lugens* through interfering with reproduction [3,4].

Based on an analysis of genome and transcriptome data, there are five *proclotting enzyme* (*PCE*) genes in *N. lugens* genome [5]. PCEs belong to the clip-domain serine protease (clip-SPs) family, which play vital roles in embryonic development and various innate immune functions in invertebrates such as antimicrobial activity, cell adhesion, hemolymph clotting, pattern recognition, and regulation of the prophenoloxidase system [6]. PCE in the horseshoe crab *Tachypleus tridentatus* was the first serine protease-containing clip domain protein identified, and the domain was named such because it can be drawn in the shape of a paper clip in a schematic form to show the disulfide linkages [7,8]. In *T. tridentatus*, PCE is involved in a coagulation cascade that has since been widely employed as a simple and very sensitive assay for endotoxins [9]. Clip-SPs have also been reported from many species, such as immune response in *Chlamys farreri* [10], antibacterial defense in *Penaeus monodon* [11], prophenoloxidase activation cascade in *Manduca sexta* [12,13,14], *Aedes aegypti* [15], *Anopheles gambiae* [16,17], *Tenebrio molitor* [18], *Holotrichia diomphalia* [19,20], *Bombyx mori* [21], and the Toll signaling pathway on dorsal-ventral axis of *Drosophila melanogaster* embryogenesis [22,23,24,25]. However, the functions of most clip-SPs are unknown, even in well studied insect species [23].

In *N. lugens*, *Nl*PCEs were considered to mediate innate immunity [5], where *Nl*PCE1 showed bacteria-induced gene expression [26]. *Nl*PCE1 therefore appears to have a role in the host defense response, but the function of other PCEs in *N. lugens* is still unclear. In our previous study of *N. lugens* ovarian transcriptome (data unpublished), we found *NlPCE3* was highly expressed in the ovary, which implied *NlPCE3* to be important in ovarian development. In the present study, *NlPCE3* was cloned and its encoded protein sequence was analysed. RNA interference (RNAi) was then used to knock down its expression, and its functions in *N. lugens* were also investigated.

## 2. Materials and Methods

### 2.1. Insect Rearing

*N. lugens* used in this study were maintained in a climatron at China Jiliang University, Hangzhou, China. The insects were reared on rice seedlings (Taichung Native 1, TN1) at 27 ± 1 °C with 60% humidity under a 16 h light: 8 h dark photoperiod.

### 2.2. Cloning the cDNA of NlPCE3

Total RNA was isolated from *N. lugens* adult females with a TaKaRa MiniBEST Universal RNA Extraction Kit (Takara, Dalian, China), according to the manufacturer’s instructions. The RNA quantity was confirmed by agarose gel electrophoresis and analysed with a NanoDrop 2000 spectrophotometer (Thermo Scientific, Waltham, MA, USA). One μg total RNA was then used to synthesize cDNA in 20 μL reactions using PrimeScript™ II 1st Strand cDNA Synthesis Kit (Takara, Dalian, China). In order to clone the *NlPCE3* sequence, a PCR primer pair for *NlPCE3* was designed with primer premier 5.0 (Table 1). Premix Taq™ (Takara, Dalian, China) was used to amplify the PCR product. PCR product was cloned into a pMD19-T vector (Takara, Dalian, China) and then sequenced by Sanger’s method.

### 2.3. Sequence Comparison and Phylogenetic Analysis

The *Nl*PCE3 protein sequence was compared with other Clip-SP sequences from a NCBI BLAST (https://blast.ncbi.nlm.nih.gov/Blast.cgi), and these sequences were aligned with ClustalW. Physical parameters and signal peptide position were predicted by ExPASy (https://web.expasy.org/protparam/) and SignalP (http://www.cbs.dtu.dk/services/SignalP/), respectively. Transmembrane helices were analysed by TMHMM Server v. 2.0 (http://www.cbs.dtu.dk/services/TMHMM-2.0/). Phosphorylated sites were predicted by KinasePhos (http://kinasephos.mbc.nctu.edu.tw/predict.php).

### 2.4. Real-time Quantitative PCR Analysis

Real-time quantitative PCR (qPCR) was carried out to analyse the expression level of target genes. Total RNA was isolated as described above. One μg RNA was used for reverse transcription in a 20 μL reaction using Perfect Real Time PrimeScript™ RT reagent Kit with gDNA Eraser (Takara, Dalian, China). qPCR was performed in triplicate on Step One plus (ABI, USA) using SYBR® Premix Ex Taq™ II (Tli RNaseH Plus) (Takara, Dalian, China). A specific primer pair for qPCR was designed and *N. lugens 18S rRNA* (*Nl18S*) was used as an internal control (Table 1). The qPCR profile was: 94 °C for 30 s followed by 40 cycles at 94 °C for 5 s and 60 °C for 30 s. The specificity of primers was confirmed by melting curve analysis and Sanger sequencing. According to the curve based on the cycle threshold and logarithm of copy concentration, the amplification efficiency of *NlPCE3* and *Nl18S* primers were calculated and they are both 103%, so the expression variation of *NlPCE3* can be evaluated by 2^-ΔΔCt^ method.

### 2.5. RNA Interference

Because the +1 to +6 region of T7 promoter is vital for the efficient synthesis of double strand RNA (dsRNA) in vitro using T7 RNA polymerase and synthetic DNA templates [27], we carefully searched the special six nucleotide bases in target gene sequences, and primers containing the T7 promoter sequence for synthesizing dsRNA were designed (Table 1). The template of *NlPCE3* for dsRNA synthesis was 630 bp (from 798 to 1427). dsRNA of *GFP* gene (ds*GFP*) was taken as the negative control. The *GFP* gene sequence was synthesized in vitro referred to the binary vector pCAMBIA-1302 (GenBank: AF234298.1), and was cloned to pMD19-T vector (Takara, Dalian, China). The template of *GFP* for dsRNA synthesis was 350 bp (from 282 to 631). The PCR amplification product of the target gene was used as the template to synthesize dsRNA using a MEGAscript T7 transcription kit (Ambion, Austin, TX, USA), according to the manufacturer’s instructions. The quality and size of the dsRNA products were verified by 1% agarose gel electrophoresis and the NanoDrop 2000 spectrophotometer. Approximately 50 nL of dsRNA (5000 ng/μL) was injected into the abdomen of each newly emerged virgin macropterous female using a manual microinjector.

### 2.6. Dissection Observation and Fertility Analysis

Insects treated with dsRNA were dissected in PBS. The dissected tissues were observed under a stereozoom microscope (Nikon SMZ1500, Tokyo, Japan), and photographed with NIS Elements software. To analyse fertility, one treated female (24 h after dsRNA treatment) was mated with two untreated males in a bottle containing fresh rice seedlings that were changed every day. Mortalities were calculated each day and the number of eggs was counted after oviposition until the females died.

### 2.7. Immunofluorescence

Immunofluorescence was performed to analyse the localization and distribution of target proteins. The ovary, fat body, and gut were dissected from macropterous females. Fixing and staining were performed as previously described [28]. Anti-*Nl*PCE3 primary rabbit polyclonal antibody was purified from New Zealand rabbits after injection of an *Nl*PCE3 polypeptide (VEGKSRHRRSIGDQ) antigen solution. The specificity of the anti-*Nl*PCE3 antibody was evaluated by western blot (Figure S1). Goat anti-rabbit IgG antibody conjugated with Dylight 488 fluorescent dye was used as a secondary antibody. The samples were observed with a laser scanning confocal microscopy (Leica SP8, Mannheim, Germany).

## 3. Results

### 3.1. Identification and Phylogenetic Analysis of NlPCE3

**Figure 1:**
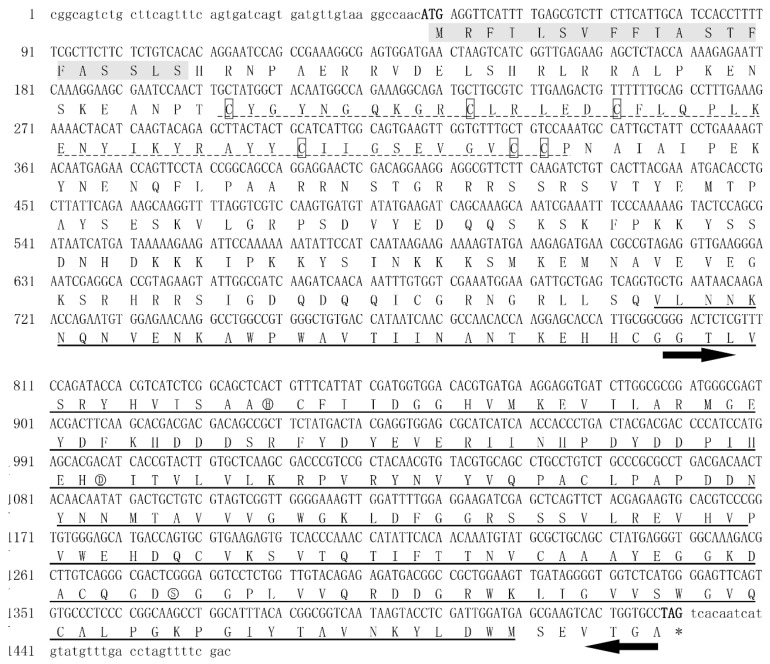
Nucleotide and deduced amino acid sequences of *NlPCE3*. The start codon (ATG) and stop codon (TAG) are displayed in bold. The numbers on the left side of the figure correspond to the nucleotide sequence. *Nl*PCE3 is composed of a signal peptide (in shaded), a clip domain (dot-lined) with six cysteine (in box), and a trypsin-like serine proteinase domain (underlined). The catalytic triad is marked with a circle (H, D, and S). The template sequence for in vitro ds*NlPCE3* synthesis was pointed (black arrow).

The ORF of *NlPCE3* potentially encodes 460 amino acids (Figure 1) (GenBank: MN586815). The predicted protein *Nl*PCE3 is deduced with a molecular mass of approximately 52.2 kDa and a calculated pI value of 8.94. *Nl*PCE3 contains a 20 amino acid N-terminal signal peptide, but no transmembrane domain. As expected, *Nl*PCE3 does contain two typical clip serine protease domains, a clip domain with six cysteines, and a trypsin-like serine protease domain with the three conserved catalytic sites (H264, D317, and S410). *Nl*PCE3 has 13 phosphorylation sites, including seven serine sites (S45, S118, S124, S125, S164, S183, and S201), two threonine sites (T119 and T124) and four tyrosine sites (Y149, Y309, Y345, and Y444).

Clip-SP sequences from several species were aligned with *Nl*PCE3 and compared. The alignment shows that all sequences have two conserved structural domains with six cysteines and three conserved catalytic sites (Figure S2). The two domains of *Nl*PCE3 exhibit high homology with those of other clip-SP sequences. However, the intervening sequence between the clip and serine protease domains in *Nl*PCE3 has low identity to that of other clip-SPs. Sequence comparisons also show that *Nl*PCE3 is 32%, 29%, 26%, and 20% identical to *Nl*PCE1, *Nl*PCE2, *Nl*PCE4, and *Nl*PCE5, respectively (Figure S3).

### 3.2. Developmental and Tissue-specific Expression of NlPCE3

**Figure 2:**
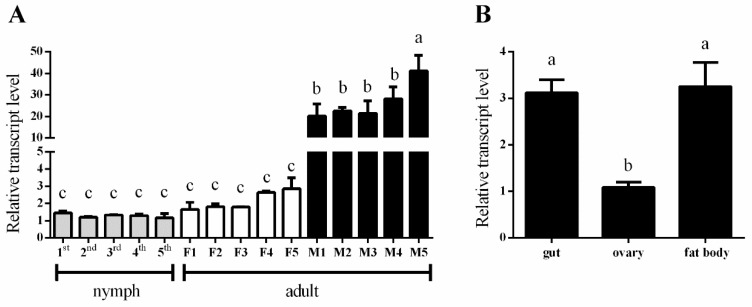
Expression of *NlPCE3* in different developmental stages and tissues. (**A**) Expression patterns of *NlPCE3* in different developmental stages, including 1^st^ to 5^th^ nymphs, adults of F (one- to five-day-old macropterous females) and M (one- to five-day-old macropterous males). (**B**) Expression patterns of *NlPCE3* in the gut, ovary, and fat body of macropterous females. Data are presented as the mean ± standard error. Different lower-case letters above the bars indicate significant differences (one way ANOVA was performed using GraphPad Prism Software 6.0, *p* < 0.05).

qPCR is used to detect the developmental and tissue-specific expression of *NlPCE3* (Figure 2). *NlPCE3* was found expressed in all developmental stages. Its expression in male adults was significantly higher than in nymphs and female adults (Figure 2A). The expression of *NlPCE3* in the gut, ovary, and fat body of adult females was also detected, and the expression was higher in the gut and fat body compared with the ovary (Figure 2B).

### 3.3. Effects of RNA Interference

**Figure 3:**
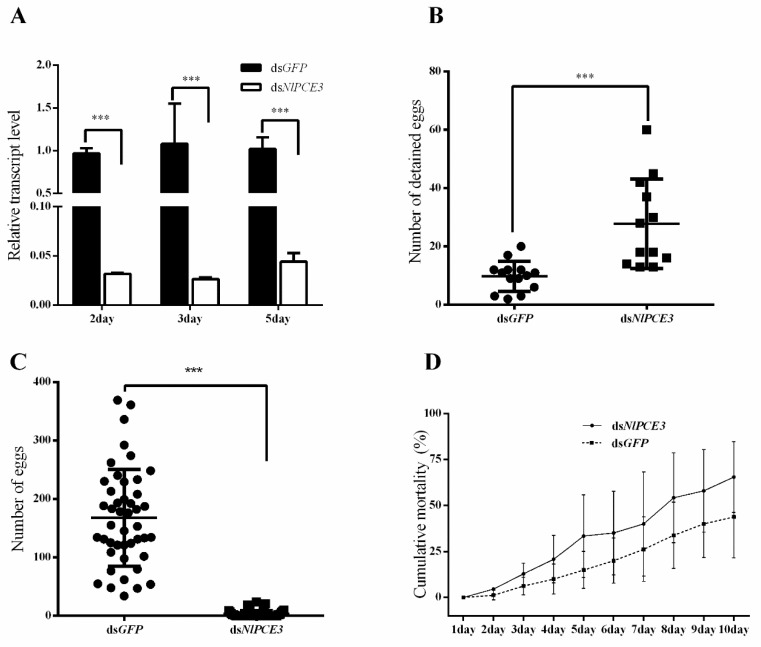
The effects of RNA interference. (**A**) Down-regulation of *NlPCE3* using ds*NlPCE3* in different days. (**B**) The number of detained eggs in female adults at 5^th^ day after injected with ds*NlPCE3* (n = 12) or ds*GFP* (n = 14). (**C**) The number of eggs laid by females injected with ds*NlPCE3* (n = 55) or ds*GFP* (n = 45). (**D**) The cumulative mortality (%) of *N. lugens* after ds*NlPCE3* and ds*GFP* injection (three groups, 20 individuals in each group). All data are presented as the mean ± standard error, one way ANOVA and unpaired two-tailed Student’s t-tests were performed using GraphPad Prism Software 6.0. *** in (**A**–**C**) represents 0.001 significance of difference. No significant difference was found in (**D**).

The expression of *NlPCE3* in whole body was notably inhibited two days (96.7%), three days (97.5%) and five days (95.6%) after *NlPCE3* dsRNA (ds*NlPCE3*) injection, compared with *GFP* dsRNA (ds*GFP*) injection (Figure 3A), indicating that ds*NlPCE3* effectively inhibits the expression of *NlPCE3*.

Eggs were detained in follicles after ds*NlPCE3* injection, while some eggs of ds*GFP*-treated individuals have been ovulated (Figure 3B). On the contrary, only a few to no eggs were laid by females after ds*NlPCE3* treatment, whereas each female laid more than 150 eggs on average after ds*GFP* treatment (Figure 3C), indicating that ds*NlPCE3* affects the oviposition of *N. lugens*.

Mortality following dsRNA treatment was also calculated (Figure 3D). The result showed that ds*NlPCE3*-treated females died at a higher rate than ds*GFP*-treated females, although the difference was not statistically significant. The difference in mortality between ds*NlPCE3*-treated and ds*GFP*-treated largely occurred during the first five days.

**Figure 4:**
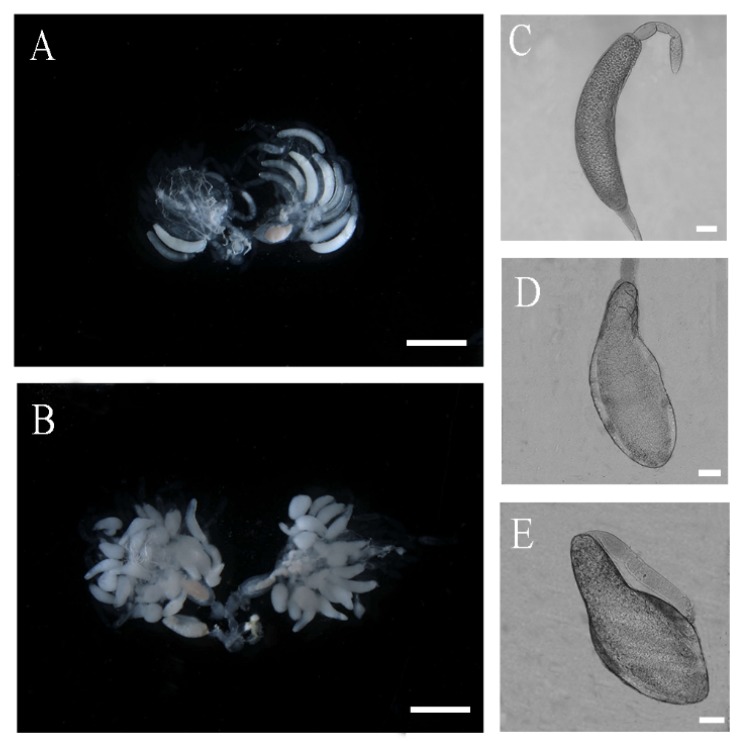
Effect of ds*NlPCE3* on *Nilaparvata lugens* ovarian development. (**A**) Ovaries of a control female adult treated at 5^th^ day after ds*GFP* injection. (**B**) Ovaries of a female adult at 5^th^ day after ds*NlPCE3* injection. (**C**) Normal mature egg. (**D**–**E**) Abnormal egg. Scale bar: 1 mm (**A**,**B**); 100 μm (**C**–**E**).

Ovarian development was affected by ds*NlPCE3* injection. Up to four days after dsRNA injection (immature stage of the ovary), most ovaries were not visibly different between ds*NlPCE3* and ds*GFP* injection under anatomic microscope, but from the 5^th^ day after dsRNA injection a difference was observable. At the 5^th^ day after dsRNA injection, in the ds*GFP* group, the terminal follicles in ovarioles presented a number of banana-shaped mature eggs (Figure 4A,C), but in the ds*NlPCE3* treatment group, malformed pear-shaped or spherical terminal follicles were observed (Figure 4B). The egg in the terminal follicles were not typically banana-shaped. The posterior part of the egg was spherical and its eggshell was not formed while at the anterior its eggshell was formed, or below the center the egg was expanded and the diameter was much bigger than the normal egg (Figure 4D,E). These results suggest that eggshell is not normally formed after ds*NlPCE3* injection.

### 3.4. Immunofluorescence Analysis of NlPCE3 Expression

**Figure 5:**
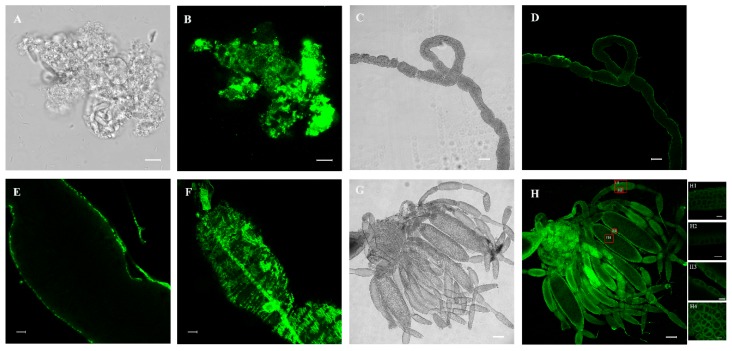
Analysis of *Nl*PCE3 expression using immunofluorescence staining. (**A**,**B**) The localization of *Nl*PCE3 in fat body. (**C**–**F**) The localization of *Nl*PCE3 in gut. (**D**–**H**) The localization of *Nl*PCE3 in ovary. H1 to H4 were respectively enlarged from H (red boxes). Scale bar: 10 μm (**A**,**B**,**E**,**F**,**H1–H4**); 100 μm (**C**,**D**,**G**,**H**).

**Figure 6:**
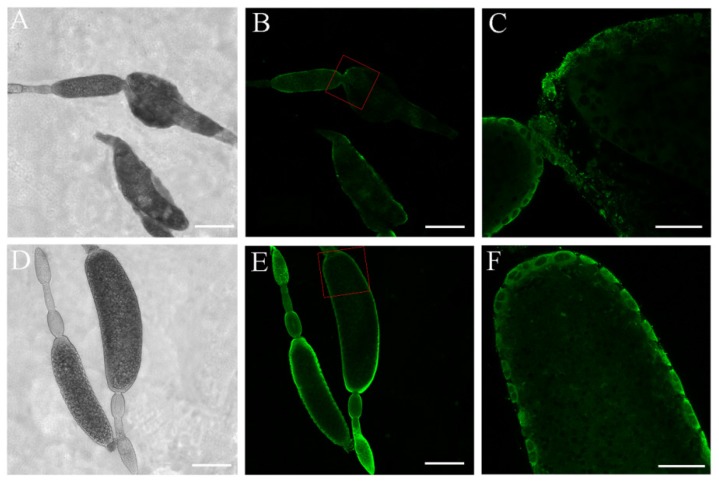
Ovarian follicle development after dsRNA treatment. (**A**,**B**) Follicles of females at 5^th^ day after ds*NlPCE3* treatment. (**C**) Enlarged from **B** (red box). (**D**–**E**) Follicles of females at 5^th^ day after ds*GFP* treatment. (**F**) Enlarged from **D** (red box). Scale bar: 200 μm (**A**,**B**,**D**,**E**); 50 μm (**C**,**F**).

To better understand the functions of *Nl*PCE3, we performed immunofluorescence staining. *Nl*PCE3 expression was observed in fat body (Figure 5 A,B), gut (Figure 5C–F), and ovary (Figure 5G,H). After ds*NlPCE3* treatment, no phenotypical change of the fat body or gut was observed. In detail, in gut epithelial cells, *Nl*PCE3 was expressed longitudinally, relative to the axis of the gut. In the ovary, *Nl*PCE3 was predominantly expressed in the cytoplasm and cytomembrane of follicular cells outside oocytes (Figure 5H and Figure 6D–F), and its expression appeared to be up-regulated with the onset of vitellogenesis (Figure 5H1–H4). Starting from the 5^th^ day after ds*NlPCE3* injection, the follicular cells outside the terminal follicle were destroyed, and the expression of *Nl*PCE3 in follicular cells was disordered (Figure 6A–C).

## 4. Discussion

In the present study, *NlPCE3* is expressed at all developmental stages and in different tissues of *N. lugens*, indicating that *NlPCE3* ubiquitously functions in *N. lugens.* Immunofluorescence staining results also show that *NlPCE3* is expressed in fat body and gut, but no obvious phenotypical change was found after ds*NlPCE3* injection. These results might be due to the followed reasons. Firstly, there might be a difference in RNAi efficiency, related to tissue-specific effort or the way of RNAi (injection or feeding). Secondly, no phenotypical change exerted after dsRNA injection in fat body and gut was possible due to compensatory/rescue mechanisms, as the *NlPCE3* expression was relatively higher in fat body and gut than ovary. Alternatively, the phenotypical changes were inconspicuous and require other technologies to explore.

The effect of *NlPCE3* RNAi on ovarian development was prominent. After treatment with ds*NlPCE3*, the follicular cells underwent degradation without clear boundaries between cells and could not maintain a regular cell layer outside terminal oocytes. Notably, *NlPCE3* RNAi caused the abnormal development of terminal follicles, the abnormal eggshell formation of terminal oocytes, and the obstruction of ovulation. These findings indicate that *NlPCE3* is necessary for normal egg development and ovulation.

Ovarial follicular cells function in patterning the oocyte, physically shaping the egg chamber, and secreting the eggshell [29]. Went and Junquera [30] demonstrated that the shape of the follicle in *Heteropeza pygmaea* was maintained by its follicular cells. In *D. melanogaster* and *Bradysia tritici*, the basement membrane of the follicular epithelium is an important factor in shaping the follicles, since cells tend to assume a spherical shape in the absence of external factors (e.g., substrate or adhesive neighboring cells) or internal forces (e.g., cytoskeleton) [31]. As reported recently, in *D. melanogaster*, the follicular cells secrete an extracellular matrix that may transform the egg chamber from spherical to elliptical [32]. In the present study, *Nl*PCE3 is widely expressed in the follicular cells. Besides, degradation of follicular cells outside the malformed terminal oocyte was found after *NlPCE3* RNAi. Degradation of follicular cells usually occurs in intact insects after movement of eggs into the lateral oviduct, but in the present study, the degradation of follicular cells occurred before the eggshell of the terminal oocytes was completely formed. Therefore, *NlPCE3* RNAi disturbed the structure intactness and function of follicular cells, and then resulted in abnormal formation of the eggshell of the terminal oocytes.

The result that ds*NlPCE3*-treated females laid much fewer eggs than ds*GFP*-treated females in this study may be due to three reasons. Firstly, because the follicular cells outside the terminal oocyte were destroyed, these cells could not support the egg to move down into the lateral oviduct of the ovary. Secondly, the abnormal eggs had a large morphology compared with the banana-shaped egg, which may arise the obstruction of ovulation. Thirdly, after treatment with ds*NlPCE3*, ovarioles were brittle in dissection, indicating the strength of the ovariole might not support the egg to move from the terminal follicle to the lateral oviduct.

Egg production is a complex activity affected by multiple factors, and some studies have tried to explore the underlined mechanism of follicular cells functioning in the process of egg production. During oogenesis of *Blattella germanica*, a calcium-binding glycoprotein, SPARC, contributes to oogenesis in panoistic ovaries by stabilizing the follicular cells program, helping to maintain the nuclear division and the cytoskeleton organization [33]. In *N. lugens*, Dicer1 helps the follicular cells providing nutrients for oocytes, and Dicer1 dsRNA treatment causes abnormal follicular cells and smaller, badly malformed oocytes [34]. In another study in *N. lugens*, the depletion of forkhead box transcription factor L2 (*NlFoxL2*) or follicle cell protein 3C (*NlFcp3C*), which is directly activated by *NlFoxL2*, suppresses the expression of genes encoding high-cysteine chorion proteins. Follicular cells can then not form proper chorion layers, resulting in spherically-shaped oocytes, obesity, and female infertility [35]. This is similar to the results of ds*NlPCE3* treatment in the present study. However, the malformation of follicles and eggs in the present study may be ascribed to the degradation of follicular cells rather than the chorion layer. Since *Nl*PCE3 has a signal peptide, it could be secreted out the follicular cells, but the result of immunofluorescence staining showed *Nl*PCE3 was predominantly expressed in the cytoplasm and cytomembrane of follicular cells. Therefore, *Nl*PCE3 possibly remains inside these cells and in membranes contributing to maintaining the stability of the follicular cells. Besides, the clip domain may have a regulatory function or may modulate interactions of proteases with their substrates or other proteins [12]. *Nl*PCE3 may therefore function in egg production by cascade regulation or covalent modification, which warrants further study.

## 5. Conclusions

In conclusion, functional analysis by RNAi revealed that *Nl*PCE3 influences the eggshell formation of eggs and ovulation, indicating that *Nl*PCE3 plays an important role in egg production in *N. lugens*. This is an important addition to the other known functions of clip-SPs. In this study, *NlPCE3* has significantly higher expression in male adults than in female adults, in accord with the previous study [5]. Additionally, *NlPCE3* is expressed higher in the gut and fat body, but no obvious phenotypical change was observed in these tissues after RNAi. These results need further studies to explain.

## Figures and Tables

**Table 1:** The primers used in this study.

Primers	Primer Sequence (5′–3′)
for cloning cDNA	
*NlPCE3*-F	CGGCAGTCTGCTTCAGTTT
*NlPCE3*-R	GTCGAAAACTAGGTCAAACAT
for qRT-PCR	
*NlPCE3*-qF	CGAAATGGAAGATTGCTGAGTC
*NlPCE3*-qR	TGGTGTTGGCGTTGATTATGG
*Nl18S*-qF	GTAACCCGCTGAACCTCC
*Nl18S*-qR	GTCCGAAGACCTCACTAAATCA
for synthesizing dsRNA	
T7-*NlPCE3*-dsF	GGATCCTAATACGACTCACTATAGGGACTCTCGTTTCCAGATACC
T7-*NlPCE3*-dsR	GGATCCTAATACGACTCACTATAGGCACCAGTGACTTCGCTC
T7-*GFP*-dsF	GGATCCTAATACGACTCACTATAGGGATACGTGCAGGAGAGGAC
T7-*GFP*-dsR	GGATCCTAATACGACTCACTATAGGGCAGATTGTGTGGACAGG

## Supplementary Materials

The following are available online at https://www.mdpi.com/2075-4450/10/11/378/s1, Figure S1: Specificity of anti-*Nl*PCE3 antibody analysed by western blot, Figure S2: Amino acid sequence comparison of clip-SPs, Figure S3: Amino acid sequence comparison of *Nl*PCE1-5.
