# Supplementary material for: A Clip Domain Serine Protease Involved in Egg Production in Nilaparvata lugens: Expression Patterns and RNA Interference
Source: Insects. 2019 Oct 30;10(11):378. doi: 10.3390/insects10110378 (PMC6920836; doi:10.3390/insects10110378)
Supplement: Supplementary file 1 [file insects-10-00378-s001.pdf]

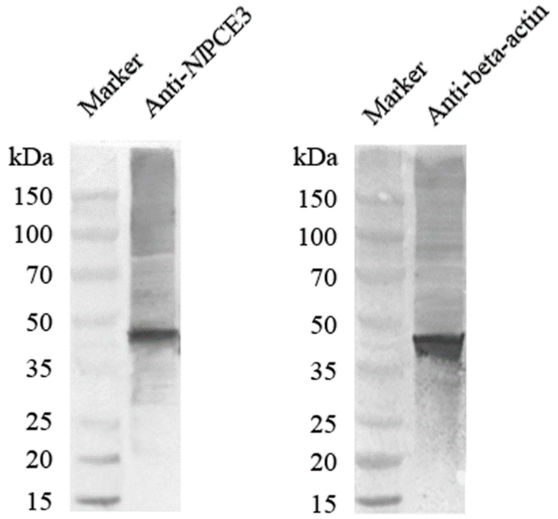

Figure S1. Specificity of anti-*Nl*PCE3 antibody analysed by western blot. Anti-*Nl*PCE3 antibody was purified from New Zealand rabbit. Anti-beta actin rabbit polyclonal antibody (Abbkine, USA) was taken as the positive control. The whole body of females was used to evaluate the specificity of the anti-*Nl*PCE3 antibody.

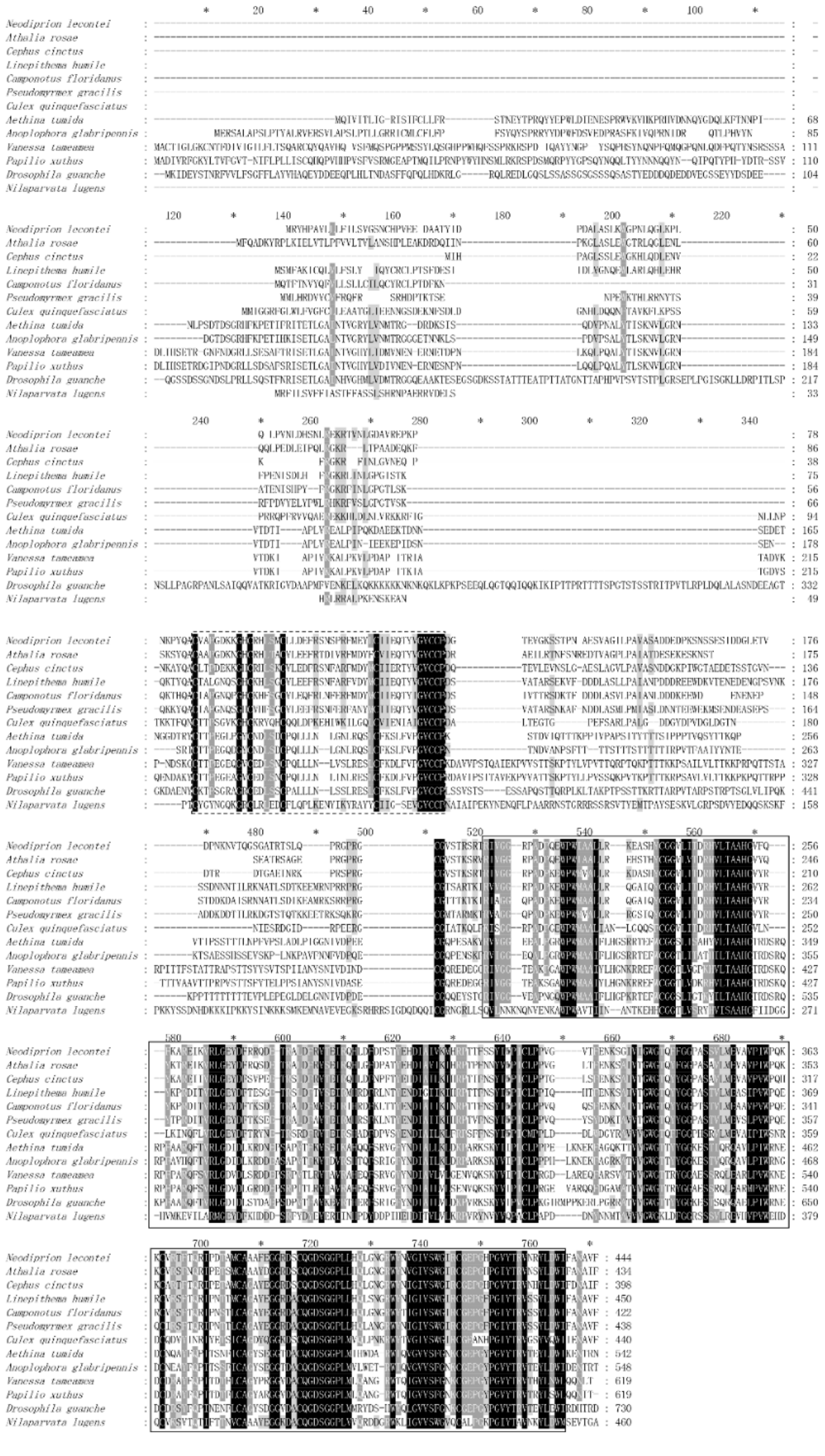
Neodiprion lecontei
Athalia rosae
Cephus cinctus
Linepithema humile
Camponotus floridanus
Pseudomyrmex gracilis
Culex quinquefasciatus
Aethina tumida
Anoplophora glabripennis
Vanessa tameamea
Papilio xuthus
Drosophila guanche
Nilaparvata lugens

Figure S2. Amino acid sequence comparison of clip-SPs. The locations of the clip domain and serine proteinase domain are in the dot-lined box and full-lined box, respectively. *Neodiprion lecontei* (XP_015524395.1), *Athalia rosae* (XP_012252741.1), *Cephus cinctus* (XP_015592174.1), *Linepithema humile* (XP_012222599.1), *Camponotus floridanus* (XP_011264758.1), *Pseudomyrmex gracilis* (XP_020295935.1), *Culex quinquefasciatus* (XP_001851428.1), *Aethina tumida* (XP_019880431.1), *Anoplophora glabripennis* (XP_018570111.1), *Vanessa tameamea* (XP_026500245.1), *Papilio xuthus* (XP_013173234.1), *Drosophila guanche* (SPP83732.1).

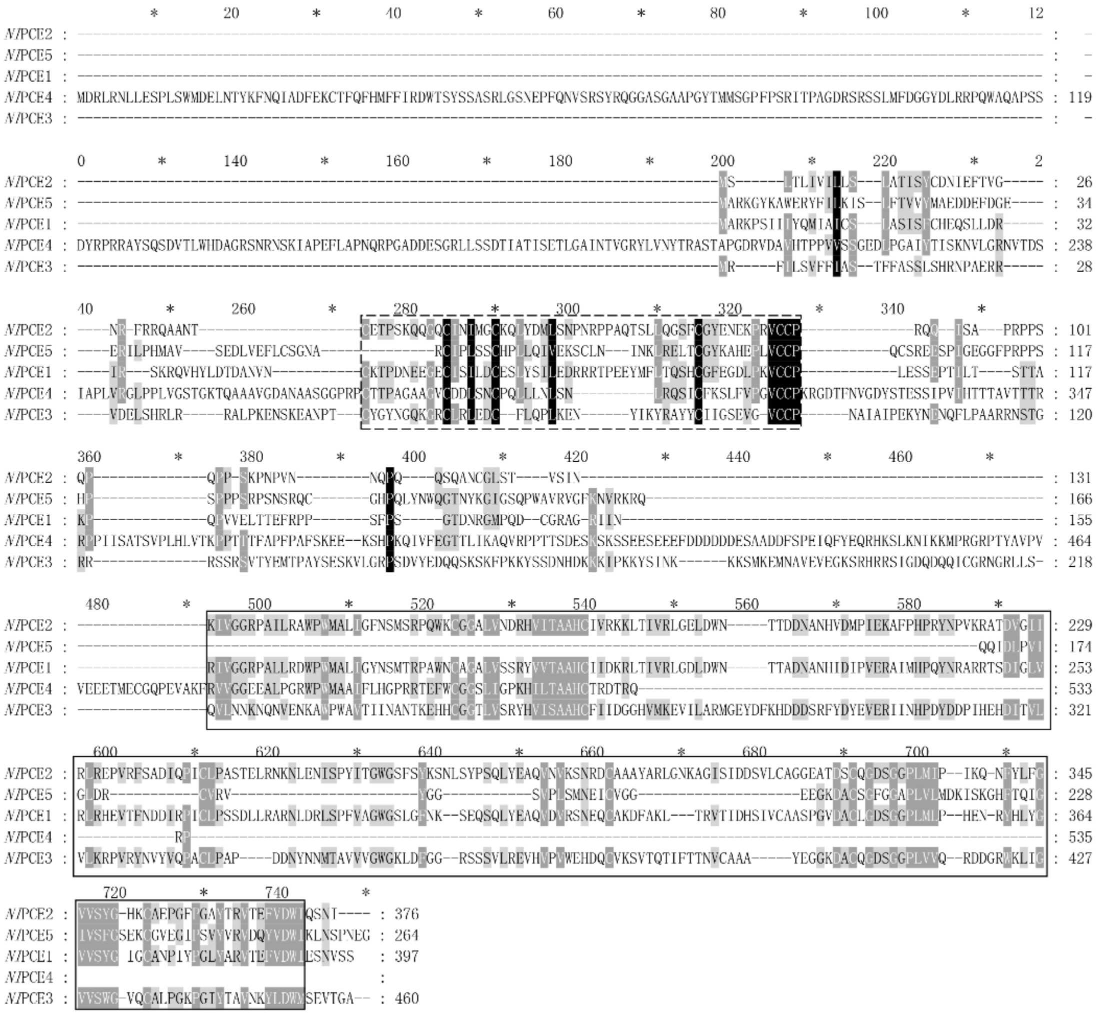

Figure S3. Amino acid sequence comparison of *Nl*PCE1-5. The locations of the clip domain and serine proteinase domain in the dot-lined box and full-lined box, respectively. *NlPCE1* (AGK40913.1), *NlPCE2* (AGK40914.1), *NlPCE4* (AGK40916.1), *NlPCE5* (AGK40917.1).
